# Primary Care Telemedicine vs In-Person Antibiotic Prescribing for Pediatric Respiratory Tract Infections

**DOI:** 10.1001/jamanetworkopen.2026.10062

**Published:** 2026-05-01

**Authors:** Kristin N. Ray, Samuel R. Wittman, Mary Kate Kelly, Janani Ramachandran, Kristin Davis, Donna Harris, Jennifer Steffes, Frances M. Biel, Everly Macario, Brigit A. Hatch, Julia E. Szymczak, Dara D. Méndez, Jonathan G. Yabes, Robert Grundmeier, Alexander G. Fiks

**Affiliations:** 1 Department of Pediatrics, University of Pittsburgh School of Medicine, Pittsburgh, Pennsylvania; 2 Children’s Hospital of Philadelphia, Philadelphia, Pennsylvania; 3 Primary Care Research, American Academy of Pediatrics, Itasca, Illinois; 4 OCHIN Inc, Portland, Oregon; 5 School of Medicine, Oregon Health & Science University, Portland; 6 Department of Internal Medicine, Spencer Fox Eccles School of Medicine, University of Utah, Salt Lake City, Utah; 7 Department of Epidemiology, University of Pittsburgh School of Public Health, Pittsburgh, Pennsylvania; 8 Department of Medicine, University of Pittsburgh School of Medicine, Pittsburgh, Pennsylvania

## Abstract

**Importance:**

Acute respiratory tract infections (ARTIs) are among the most common reasons for pediatric primary care visits and antibiotic receipt. Telemedicine outside of primary care settings has been associated with overuse of antibiotics for ARTIs in children. The quality of telemedicine when integrated within primary care for children is not clear.

**Objective:**

To compare antibiotic management during primary care visits conducted through telemedicine vs in-person.

**Design, Setting, and Participants:**

This retrospective, cross-sectional study analyzed visits for ARTIs in children younger than 18 years between January 1 and December 31, 2023, at 694 US primary care practices (including community health organizations, independent pediatric practices, and practice networks affiliated with large health systems). Analyses were performed between October 1, 2024, and February 12, 2026.

**Exposure:**

Primary care telemedicine vs primary care in-person index visits.

**Main outcomes and measures:**

The primary outcomes were percentage of index visits with antibiotics prescribed and percentage with antibiotic management concordant with guidelines for visit diagnosis. Secondary outcomes included index visit diagnosis, follow-up visits within 14 days, and antibiotic prescription within 14 days. Weighted analyses were conducted using a propensity score model to estimate the probability of an ARTI index visit being conducted via telemedicine and estimated the average treatment effect associated with telemedicine.

**Results:**

This study included 438 148 in-person and 11 482 telemedicine index ARTI visits at primary care practices by 302 817 children (mean [SD] age: 6.6 [4.7] years; 51.4% male). Antibiotic prescription occurred during 46.8% (95% CI, 45.1%-48.4%) of primary care in-person visits vs 34.6% (95% CI, 27.0%-42.3%) of primary care telemedicine visits in the propensity score–weighted model, with a difference of −12.1 (95% CI, −19.3 to −5.0) percentage points. Antibiotic management was guideline concordant for 86.2% (95% CI, 85.1%-87.3%) of primary care in-person visits vs 85.5% (95% CI, 80.5%-90.4%) of primary care telemedicine visits, with a difference of −0.7 (95% CI, −5.3 to 3.8) percentage points. The proportion of follow-up visits and antibiotic prescription within 14 days after initial visit did not vary significantly by index visit modality.

**Conclusions and Relevance:**

In this cross-sectional study of primary care practices caring for children, telemedicine integrated within primary care was associated with judicious antibiotic prescribing without increased follow-up visits or subsequent antibiotics prescribed. Supporting primary care practices in offering telemedicine for acute concerns may be a strategy to limit unnecessary antibiotic receipt.

## Key Points

**Question:**

How does antibiotic management for pediatric acute respiratory tract infections compare during primary care visits conducted through telemedicine vs in person?

**Findings:**

In this cross-sectional analysis of 694 US pediatric and family medicine primary care clinics, antibiotic prescription occurred in 34.6% of primary care telemedicine visits vs 46.8% of primary care in-person visits, a significant difference. The percentage of visits with antibiotic management that aligned with the given diagnosis did not vary significantly by visit modality.

**Meaning:**

These findings suggest that telemedicine visits integrated within primary care for children were associated with less antibiotic prescribing without increased follow-up visits or subsequent antibiotic prescriptions, compared with in-person visits.

## Teaser

This cross-sectional study assesses how antibiotic prescription and management for pediatric acute respiratory tract infections compare during telemedicine and in-person primary care visits.

## Introduction

Acute respiratory tract infections (ARTIs) are a cluster of diagnoses, including viral upper respiratory tract infections, acute otitis media, streptococcal pharyngitis, and bacterial sinusitis, which can present with overlapping symptoms, such as cough, congestion, sore throat, earache, and fever. In pediatric practice, ARTIs constitute up to 20% of pediatric problem–based visits^1^ and account for the majority of pediatric outpatient antibiotic prescriptions.^2^ In both primary care and emergency settings, more than 50% of visits for ARTIs result in antibiotic prescriptions.^3,4^ Prior studies estimate that between one-quarter and one-half of antibiotics prescribed for children with respiratory concerns are unnecessary.^3,5^ For families, ARTIs also contribute to psychological impacts such as worry and economic impacts such as missed work.^6^

Given the high burden of ARTI visits for children and families, understanding whether and how telemedicine can deliver high-quality care to children with ARTI symptoms is important. Telemedicine has the potential to reduce travel and time burden for families seeking ARTI care, but these benefits must be weighed against measures of quality and the potential harms of misdiagnoses, overtreatment, or undertreatment.^6,7^ For ARTIs, key measures of quality include rates of overall antibiotic prescribing and alignment of antibiotic management with published guidelines for the visit diagnosis.^8,9,10^ Additionally, rates of follow-up visits and subsequent antibiotic prescriptions are measures that may indicate missed diagnoses or complications of treatment.

In previous analyses, emerging direct-to-consumer telemedicine models of care (in which care is delivered by a commercial vendor outside of a child’s usual primary care practice)^11^ were associated with higher rates of antibiotic receipt and lower rates of guideline-concordant care than in-person primary care or urgent care visits.^12^ Since 2020, policy and regulatory changes have afforded primary care practices greater ability to receive payment for telemedicine visits, allowing for the investigation of the quality of care when primary care practices use telemedicine to care for pediatric ARTIs. Early post–COVID-19 pandemic studies indicated the potential for high guideline concordance in primary care telemedicine visits within 1 practice network,^13^ with a subsequent study reporting decreased antibiotic prescribing for primary care telemedicine compared with direct-to-consumer telemedicine.^14^ These studies raise questions about the outcomes of primary care telemedicine ARTI visits within a broader sample of primary care practices, including pediatric and family medicine clinics.

To characterize antibiotic prescribing for ARTIs during telemedicine integrated within primary care practices, we examined electronic health record (EHR) data from 694 US primary care practices, including community health organizations, independent pediatric practices, and 2 pediatric practice networks affiliated with large health systems. Specifically, we compared antibiotic prescribing and guideline-concordant management for telemedicine visits compared with in-person visits. We examined these outcomes across the full sample and stratified by patient and visit demographics to understand whether telemedicine is associated with different quality overall or for any patient subgroups.

## Methods

### Study Setting

This study was a retrospective, cross-sectional analysis of primary care visits between January 1 and December 31, 2023, in an EHR-derived limited dataset, the Telemedicine Integrated into Pediatric Primary Care dataset. This dataset harmonized data contributed from multiple existing research and practice networks, including the Pediatric Research in Office Setting network, OCHIN, the University of Pittsburgh Medical Center, and the Children’s Hospital of Philadelphia, thereby including US pediatric and family medicine primary care practices from a national network of community health organizations, a national network of independent pediatric practices, and 2 regional pediatric practice networks affiliated with large health systems. In this analysis, we included family medicine and pediatric primary care practices with at least 100 visits by children during the study year and nonzero use of telemedicine. The included 694 practices were located in 45 US states and the District of Columbia. The American Academy of Pediatrics Institutional Review Board provided human participant approval, including waiving informed consent for this limited dataset study of deidentified data. We used the Strengthening the Reporting of Observational Studies in Epidemiology (STROBE) guideline for reporting.^15^

### Identifying ARTI Visits and Episodes

We identified telemedicine and in-person visits by children younger than 18 years with bacterial and viral ARTI diagnoses based on *International Classification of Disease and Related Health Problems*, *Tenth Revision* (*ICD-10*) codes as in prior studies (eTable 1 in Supplement 1).^13,14,16,17,18,19^ We focused on visits with ARTI diagnoses (as opposed to visits with *ICD-10* codes for symptoms [eg, cough] but no diagnosis) due to prior analyses showing minimal difference in results from including such visits.^14^

From these ARTI visits, we constructed episodes of care by identifying index visits with no ARTI visit within the previous 21 days. For each index visit, any visits within the subsequent 14 days were associated with that index visit as follow-up visits within an episode of care.^14^ We excluded ARTI episodes with a diagnosis of pneumonia, bronchiolitis, COVID-19, or influenza because our prior mental models study indicated that parental concern about respiratory distress or desire for viral testing prompt different parent care-seeking decisions (and different likelihood for use of telemedicine).^6^ We then excluded episodes that began with an index visit coded as a well child visit (because well child visits were almost entirely conducted in-person) or that had a codiagnosis warranting antibiotics (eg, urinary tract infection) based on *ICD-10* codes used in prior studies.^19^

### Exposure

We identified telemedicine visits based on the presence of billing code modifiers GT or 95, billing place of service codes, or EHR scheduling data indicating a video visit type or activation of video during the visit, drawing from prior studies validating identification of telemedicine visits,^20,21^ with modifications to account for evolving billing requirements and available metadata. These data could not reliably distinguish audio-only from audio-video telemedicine.^21^

### Outcomes

Primary outcomes of interest were (1) receipt of any systemic antibiotic prescription and (2) receipt of guideline-concordant antibiotic prescriptions based on visit diagnosis. Any systemic antibiotic prescription within 2 days after an index visit was linked to that visit as in prior studies and to allow for watchful waiting. ^14^ Guideline concordance of antibiotic management was determined based on diagnosis: for streptococcal pharyngitis, amoxicillin or penicillin was concordant; for acute otitis media or sinusitis, amoxicillin, amoxicillin-clavulanic acid, or no systemic antibiotic was concordant; for viral infections, no systemic antibiotic was concordant.^8,9,10,22^ As a secondary outcome, we examined diagnosis mix at each site because diagnosis is susceptible to diagnosis shifting, in which bacterial diagnoses are selected to avoid identification of antibiotic prescribing as inappropriate.^23,24^ To capture potential downstream consequences of underdiagnosis or undertreatment, we also examined follow-up ARTI primary care visits (in-person or telemedicine visits) within the 14 days after the index visit and antibiotics prescribed for ARTI diagnoses within the 14 days after the index visit.

### Covariates

We derived covariates from EHR data, including child’s race and ethnicity (categorized as Black, Hispanic, White, or other [as per each EHR’s documentation and practice’s workflow]), child’s age, census division (categorized into 9 US regions), visit seasonality, clinic specialty (categorized as pediatrics vs family medicine), child medical complexity (using the pediatric complex chronic classification system^25^), child insurance payer (categorized as Medicaid, other payer [eg, commercial], or uninsured), child family language (categorized as English, Spanish, or other language [eg, Arabic]), and child urban or rural status (categorized as urban area, micropolitan area, or small town or rural). Children identified as other race in this analysis included those whose race in the EHR was listed as American Indian or Alaska Native, Asian, Native Hawaiian or Other Pacific Islander, multiracial, or unknown. We recognize limitations in the reporting of variables such as race, ethnicity, and family language in EHR data, and we acknowledge that race is a social construct. We include race and ethnicity in this analysis because of the potential for racism to influence both access to and quality of care.

### Statistical Analysis

Analyses were performed between October 1, 2024, and February 12, 2026. We created a propensity score model to estimate the probability of an ARTI index visit occurring via telemedicine. The propensity score represents the conditional probability that a child received telemedicine vs in-person care given their baseline characteristics. Using inverse probability of treatment weights (IPTW), we weighted each observation by the inverse of the probability of receiving the visit modality they actually received. This weighting creates a pseudopopulation in which baseline characteristics are balanced between treatment groups, therefore adjusting for measured confounding. To do this, we performed logistic regression with index visit telemedicine usage as the outcome, a practice-level random intercept to account for potential correlation within practice, and additional covariates selected based on theoretical or empirical association with antibiotic prescribing or guideline concordance. Specifically, we included child’s race and ethnicity, child’s age, census division, visit seasonality, clinic specialty, child medical complexity (using individual organ system indicators), and health care use in the previous 12 months (well child visits, problem visits, telemedicine visits, and antibiotics prescribed).^26,27,28,29,30^ We generated the model-predicted probability that each index visit occurred via telemedicine.^31^ We then calculated and applied IPTW, thereby creating a reweighted sample in which the distributions of covariates were balanced between the telemedicine and in-person groups.^32^

We assessed covariate balance using Love plots of the absolute standardized bias (ASB) before and after applying weights.^31,32^ While we initially aimed to achieve a strict ASB less than 0.1 for all covariates, we adopted a more pragmatic and commonly accepted threshold, considering ASB values approaching 0.25 to indicate reasonable balance for complex, real-world EHR data. To further assess balance, we computed the prognostic score and examined the preweighting and postweighting distributional balance and ASB between groups.^33^ After identifying poor balance for region and season covariates in our initial model, we tested an interaction term between season and region and observed improved postweighting balance. We also tested the addition of patient-level random effects, which had minimal impact on estimates and were not included in the final propensity score model. We excluded 11.9% of ARTI episodes due to missing census division or race and ethnicity data; excluded episodes were similar to included episodes on observed characteristics. Missingness for other descriptive variables is detailed in Table 1, but these variables were not included in the propensity score model and did not impact analysis.

**Table 1.** Acute Respiratory Tract Infection (ARTI) Episode-Level Covariate Distribution and Balance Measures Before and After Applying Inverse Probability of Treatment Weights (IPTW)

Covariate	Before IPTW applied^a^			After IPTW applied^a^		
	ARTI visits, No. (%)		ASB	ARTI visits, weighted %		ASB
	In-person	Telemedicine		In-person	Telemedicine	
Unweighted No.	438 148	11 482	NA	438 148	11 482	NA
Age group, y						
<12	369 524 (84.3)	9085 (79.1)	−0.14	84.2	83.0	−0.03
≥12	68 624 (15.7)	2397 (20.9)	0.14	15.8	17.0	0.03
Season						
Spring	103 532 (23.6)	2464 (21.5)	−0.05	23.6	24.5	0.02
Summer	68 401 (15.6)	1597 (13.9)	−0.05	15.6	15.0	−0.02
Fall	135 078 (30.8)	3041 (26.5)	−0.1	30.7	37.1	0.14
Winter	131 137 (29.9)	4380 (38.1)	0.17	30.1	23.4	−0.14
Census region						
Northeast	258 396 (59.0)	1653 (14.4)	−1.04	57.8	56.8	−0.02
Midwest	41 641 (9.5)	1178 (10.3)	0.03	9.5	11.5	0.07
South	68 021 (15.5)	1000 (8.7)	−0.21	15.4	12.7	−0.08
West	70 090 (16.0)	7651 (66.7)	1.2	17.3	18.9	0.04
Race and ethnicity						
Black	43 278 (9.9)	1154 (10.0)	<0.01	9.9	9.4	−0.02
Hispanic	91 592 (20.9)	6618 (57.6)	0.81	21.8	21.6	<-0.01
White	278 083 (63.5)	2398 (20.9)	−0.96	62.4	64.3	0.04
Other^b^	25 195 (5.8)	1313 (11.4)	0.2	5.9	4.7	−0.04
Medical complexity	59 329 (13.5)	1976 (17.2)	0.1	13.6	13.3	<-0.01
Clinician specialty						
Pediatrics	369 138 (84.2)	4284 (37.3)	−1.1	83.1	82.9	−0.01
Family medicine	69 010 (15.8)	7198 (62.7)	1.1	16.9	17.1	0.01
Health care use in past 12 mo						
Any well visits	199 925 (45.6)	3596 (31.3)	−0.3	45.3	44.9	−0.01
Any problem visits	249 546 (57.0)	6414 (55.9)	−0.02	57.0	65.3	0.17
Any telemedicine use	17 231 (3.9)	3699 (32.2)	0.79	4.7	8.0	0.09
Any systemic antibiotics	103 288 (23.6)	922 (8.0)	−0.44	23.2	32.5	0.26

Abbreviations: ASB, absolute standardized bias; NA, not applicable.

^a^ ARTI episode characteristics before and after IPTW are applied. Residence in 1 of 9 census divisions was entered into the model but were collapsed into the 4 census regions for data presentation. This final propensity score model achieved well-balanced characteristics, with ASB ≤ 0.10 for 30 of 36 covariates and excellent prognostic score balance (ASB = 0.06), thereby indicating adequate balance.

^b^ Children identified as other race in this analysis included those whose race in the electronic health record was listed as American Indian or Alaska Native, Asian, Native Hawaiian or Other Pacific Islander, multiracial, or unknown.

We applied final IPTWs to calculate weighted averages by visit modality (telemedicine vs in-person) for primary outcomes (antibiotic prescription and guideline-concordant antibiotic management) as well as for diagnoses received, follow-up visits, and subsequent antibiotics. We calculated the average treatment effect (ATE) associated with telemedicine use using a nonparametric marginal estimator,^34^ which estimates the weighted averages in the telemedicine and in-person groups. We calculated Wald 95% CIs for these weighted averages and ATE estimates using robust SEs, accounting for weighting and clustering within practices.

We then investigated the impact of telemedicine within patient subgroups selected due to association with differences in use or outcomes of pediatric care, including race and ethnicity, child age, family language, urban or rural setting, insurance payer, health system type, and clinician specialty.^27,28,35,36^ We generated a propensity score model within each subgroup to balance other covariates, then repeated the analysis above to obtain ATE estimates and 95% CIs within each subgroup. As a sensitivity analysis to downweigh individuals with extreme propensity scores and focus on the region of best overlap, we used overlap weights, thereby estimating the average treatment effect in the overlap population (ATO). In additional sensitivity analysis to estimate the effect of treatment modality conditional on having received a specific diagnosis, we limited the sample to index visits with each set of diagnoses and repeated the main analysis for the 2 primary outcomes.

We used a statistical significance level of 2-sided *P* < .05, which corresponds to a 95% CI for an ATE estimate that does not intersect zero. We used R software, version 4.4.0 (R Foundation), including the R dplyr package for data manipulation,^37^ the R cobalt package to assess covariate balance before and after applying IPTW,^31^ and the R survey package to generate estimates and CIs, after applying IPTW weights.^38^ SAS, version 9.4 (SAS Institute Inc) was used for the propensity score logistic regression models.

## Results

**Figure 1.**
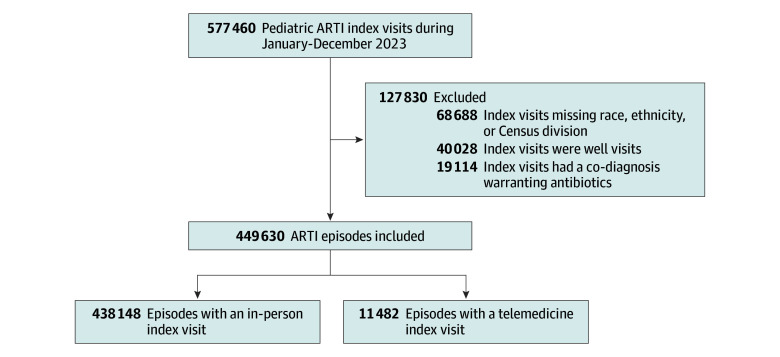
Flow Diagram of Acute Respiratory Tract Infection (ARTI) Visit and Episode Identification ARTI episodes consist of an ARTI index visit (an ARTI visit with no ARTI visit in the previous 21 days) and any ARTI visits that occurred in the subsequent 14 days.

We included 449 630 ARTI index visits among 302 817 children (mean [SD] age, 6.6 [4.7] years; 48.6% female; 51.4% male), with 438 148 in-person index visits and 11 482 telemedicine index visits (Figure 1). Before weighting, telemedicine visits included a disproportionate number of visits occurring in the West Census region, among Hispanic children, and with family medicine clinicians (ASB > 0.25 for each covariate) (Table 1). After applying IPTW, we achieved well-balanced characteristics, with an ASB less than or equal to 0.10 for 30 of 36 covariates (Table 1; eFigure 1 in Supplement 1) and excellent propensity score balance (ASB = 0.05) (eFigure 2 in Supplement 1) and prognostic score balance (ASB = 0.06) (eFigure 3 in Supplement 1), indicating adequate balance overall.

### Index Visit Outcomes

Among weighted ARTI index visits completed in person within primary care practices, 46.8% (95% CI, 45.1%-48.4%) were prescribed antibiotics (Table 2). Among ARTI index visits completed through telemedicine at a primary care practice, antibiotic prescription was lower, with 34.6% (95% CI, 27.0%-42.3%) receiving antibiotics, yielding a difference of −12.1 (95% CI, −19.3 to −5.0) percentage points.

**Table 2.** Weighted Percentages and Estimated Average Treatment Effect (ATE) of Primary Care Acute Respiratory Tract Infection (ARTI) Index Visit Outcomes by Visit Modality

Outcome	ARTI visits, weighted % (95% CI)^a^		ATE, percentage points (95% CI)^b^
	In-person	Telemedicine	
Unweighted No.	438 148	11 482	NA
Primary outcomes			
Prescribed antibiotics	46.8 (45.1 to 48.4)	34.6 (27.0 to 42.3)	−12.1 (−19.3 to −5.0)
Prescribed guideline-concordant antibiotic management	86.2 (85.1 to 87.3)	85.5 (80.5 to 90.4)	−0.7 (−5.3 to 3.8)
Secondary outcomes			
Received diagnosis potentially warranting antibiotics	44.4 (42.8 to 46.0)	33.1 (25.9 to 40.2)	−11.3 (−18.2 to −4.5)
Received diagnosis			
Streptococcal pharyngitis	13.2 (12.5 to 13.9)	7.3 (4.2 to 10.4)	−5.9 (−9.1 to −2.7)
Acute otitis media	26.3 (25.3 to 27.3)	11.0 (7.9 to 14.1)	−15.3 (−18.5 to −12.1)
Bacterial sinusitis	4.9 (4.1 to 5.6)	14.7 (9.0 to 20.5)	9.9 (4.2 to 15.6)
Viral ARTI	55.6 (54.0 to 57.2)	66.9 (59.8 to 74.1)	11.3 (4.5 to 18.2)

Abbreviations: IPTW, inverse probability of treatment weights; NA, not applicable.

^a^ Index visit outcomes for ARTI care episodes that begin with an in-person vs telemedicine index visit, after applying IPTW.

^b^ ATE is calculated as the difference in weighted percentages between the outcome for telemedicine vs in-person episode of care.

Antibiotic management was guideline concordant for 86.2% (95% CI, 85.1%-87.3%) of primary care in-person visits, which did not differ significantly from 85.5% (95% CI, 80.5%-90.4%) of primary care telemedicine visits, with a difference of −0.7 (95% CI, −5.3 to 3.8) percentage points.

Receipt of a viral diagnosis occurred at 55.6% (95% CI, 54.0%-57.2%) of in-person visits compared with 66.9% (95% CI, 59.8%-74.1%) of telemedicine visits (difference of 11.3 percentage points; 95% CI, 4.5-18.2 percentage points). Compared with in-person visits, telemedicine visits were associated with decreased diagnosis of acute otitis media (difference of −15.3 percentage points; 95% CI, −9.1 to −2.7 percentage points) and increased diagnosis of sinusitis (difference of 9.9 percentage points; 95% CI, 4.2-15.6 percentage points).

### Follow-Up Outcomes

The proportion of follow-up primary care ARTI visits within the subsequent 14 days did not differ significantly by visit modality (difference of 5.9 percentage points; 95% CI, −0.01 to 11.8 percentage points) (Table 3). The proportion of subsequent antibiotic prescription within 14 days also did not significantly different by visit modalities (difference of −0.7 percentage points; 95% CI, −2.6 to 1.1 percentage points).

**Table 3.** Weighted Percentages and Estimated Average Treatment Effect (ATE) of Primary Care Acute Respiratory Tract Infection (ARTI) Follow-Up Outcomes by Visit Modality

Outcome	ARTI visits, weighted % (95% CI)^a^		ATE, percentage points (95% CI)^b^
	In-person	Telemedicine	
Unweighted No.	438 148	11 482	NA
Subsequent primary care visits			
Within 0-2 d	1.4 (1.3 to 1.4)	5.8 (0.4 to 11.2)	4.5 (−0.9 to 9.8)
Within 3-10 d	4.9 (4.7 to 5.1)	6.9 (3.6 to 10.1)	2.0 (−1.3 to 5.3)
Within 11-14 d	2.8 (2.7 to 3.0)	2.2 (0.3 to 4.1)	−0.6 (−2.5 to 1.3)
Within 0-14 d	8.6 (8.3 to 9.0)	14.5 (8.4 to 20.6)	5.9 (−0.01 to 11.8)
Subsequent antibiotics			
Within 3-10 d	2.2 (2.1 to 2.4)	2.3 (0.6 to 4.1)	0.1 (−1.7 to 1.8)
Within 11-14 d	1.4 (1.3 to 1.5)	0.6 (−0.2 to 1.3)	−0.8 (−1.6 to −0.1)
Within 3-14 d	3.7 (3.4 to 3.9)	2.9 (1.1 to 4.8)	−0.7 (−2.6 to 1.1)

Abbreviations: IPTW, inverse probability of treatment weights; NA, not applicable.

^a^ Follow-up–related outcomes for ARTI care episodes that begin with an in-person vs telemedicine index visit, after applying IPTW.

^b^ ATE is calculated as the difference in weighted percentages between the outcome for telemedicine vs in-person episode of care.

### Subgroup Analyses

**Figure 2.**
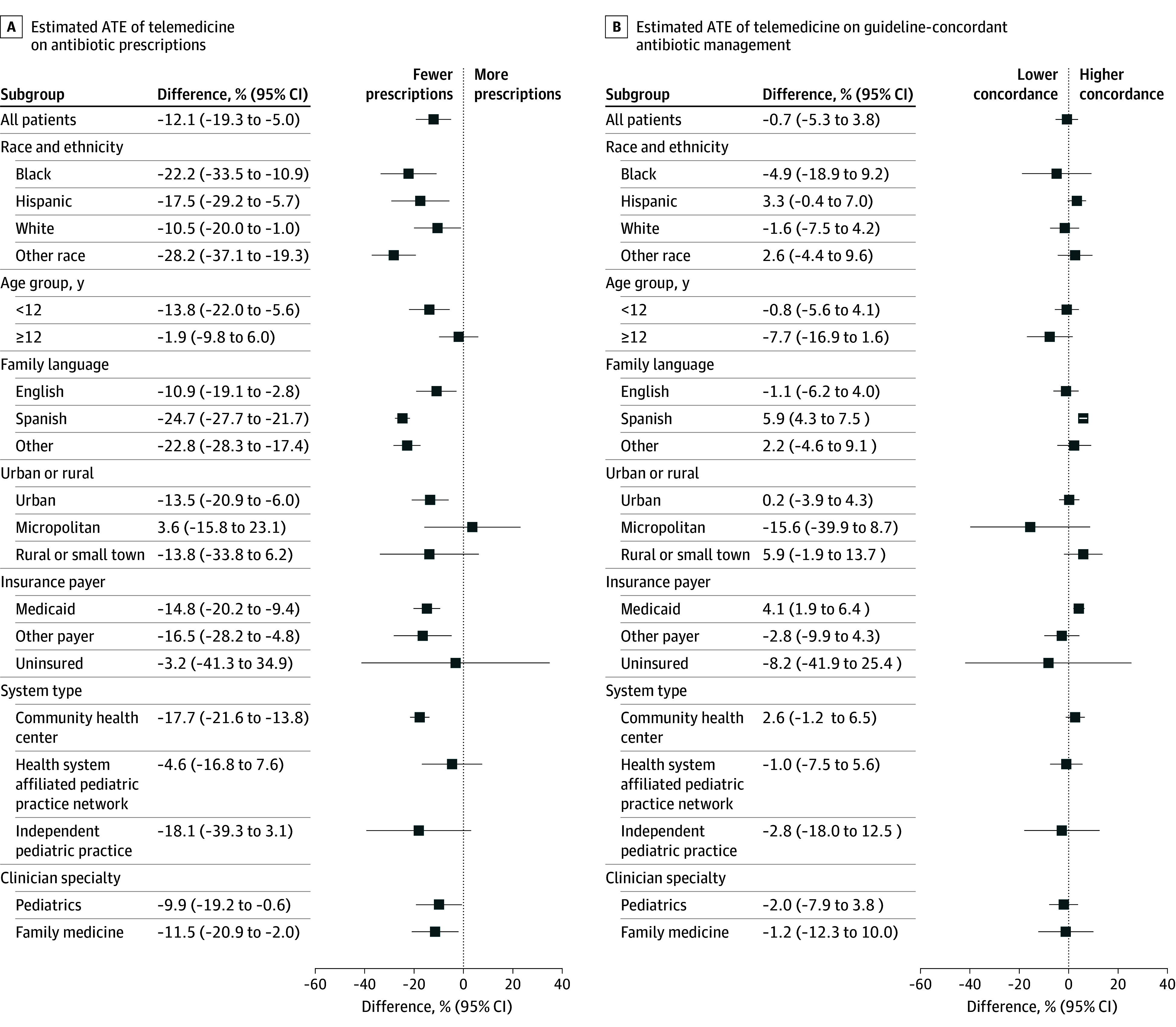
Forest Plots of Estimated Average Treatment Effect (ATE) of Primary Care Telemedicine Compared With In-Person Primary Care Visits In the panels, ATE estimates the difference in weighted percentages for outcomes associated with primary care received via telemedicine compared with in-person visits, focusing on the percentage of antibiotic prescriptions (A) and guideline-concordant antibiotic management for the visit diagnosis (B). Other race includes children whose race in the electronic health record was listed as American Indian or Alaska Native, Asian, Native Hawaiian and Other Pacific Islander, multiracial, or unknown. Other insurance payers include commercial payers. 95% CIs are shown.

Subgroup analysis achieved well-balanced postweighting propensity score distributions (eFigure 4 in Supplement 1), with results qualitatively similar to the main effects (Figure 2). In subgroup analysis focused on antibiotic prescription, there were no subgroups in which primary care telemedicine was associated with higher prescription of antibiotics than in-person visits. In subgroup analyses focused on guideline-concordant antibiotic management, telemedicine was associated with increased guideline concordance relative to in-person visits for Spanish-speaking families (difference of 5.9 percentage points; 95% CI, 4.3-7.5 percentage points) and Medicaid-insured children (difference of 4.1 percentage points; 95% CI, 1.9-6.4 percentage points). Within other subgroups, no significant difference in guideline concordance was observed by modality.

### Sensitivity Analysis

Results using overlap weights to estimate the ATO were similar to our main results (difference of antibiotic receipt: −17.7 [95% CI, −20.4 to −14.9] percentage points; difference of guideline concordance: 1.8 [95% CI, −0.2 to 3.9] percentage points).

In additional sensitivity analysis to estimate the effect of treatment modality conditional on having received a specific diagnosis, telemedicine was associated with decreased antibiotic prescribing for visits with diagnosis of streptococcal pharyngitis (difference of −18.8 percentage points; 95% CI, −28.9 to −8.6 percentage points) and acute otitis media (difference of −20.3 percentage points; 95% CI, −30.6 to −10.0 percentage points) and slightly increased antibiotic prescribing for visits with a diagnosis of sinusitis (difference of 1.6 percentage points; 95% CI, 0.4-2.8 percentage points) (eTable 2 in Supplement 1). Telemedicine visits were associated with decreased guideline concordance for visits with a diagnosis of streptococcal pharyngitis (difference of −17.3 percentage points; 95% CI, −31.0 to −3.7 percentage points) with no significant difference in guideline concordance for other diagnoses.

## Discussion

In this retrospective, cross-sectional study, we found that among patients presenting to primary care for ARTI care, receiving care through telemedicine was associated with 12-percentage-point-lower antibiotic prescribing compared with primary care in-person visits and no significant difference in guideline concordance of antibiotic management. Subgroup analyses focusing on patient demographics, geography, payer, and clinic type were similar in general to the main findings. Even though we found a lower proportion of antibiotic prescriptions at initial visits when conducted via telemedicine, we did not observe any increases in subsequent primary care visits or subsequent antibiotics, which could have indicated an increased proportion of missed diagnoses at the index visit. Our subgroup analyses also identified decreased antibiotic prescription through telemedicine visits across groups defined by child race and ethnicity, age, language, payer, and location. While the point estimate of subgroup analyses varied some, none of the subgroups showed increased antibiotic prescribing associated with primary care telemedicine. Altogether, these results indicate judicious antibiotic prescribing during ARTI telemedicine visits integrated within primary care practices of varied types and location across the US.

These results build on prior studies that examined antibiotic prescribing in a different model of telemedicine: virtual-only direct-to-consumer commercial vendors. In these prior studies, virtual-only direct-to-consumer telemedicine models (which do not allow for seeing patients in person) have been associated with increased antibiotic receipt for children relative to in-person care.^12,39^ Our findings show the opposite result for telemedicine when integrated within primary care, with antibiotic prescribing during these visits lower than in-person visits. Several factors could contribute to this finding. In contrast to virtual-only models, telemedicine integrated in primary care is the use of telemedicine by the same practice and clinicians that the patient connects with when they receive in-person care. This care delivery context is different in ways that might promote more judicious antibiotic prescribing. For children, receiving telemedicine visits conducted by their primary care practice means they are receiving care from clinicians who regularly care for children and with whom they have a preexisting relationship. Clinician training, affiliation, and continuity with patients have been associated with better prescribing practices.^26,40,41^ These factors may affect a clinician’s knowledge of and skill in diagnosing, managing, and communicating about ARTIs in children. Such knowledge and skills have proven key to promoting judicious antibiotic prescribing.^19^ In comparison with virtual-only direct-to-consumer models, telemedicine practiced in the primary care setting also has a mechanism by which a clinician can convert a telemedicine visit to an in-person visit or coordinate timely re-evaluation if necessary. This outcome could potentially reduce just-in-case antibiotic prescribing, in which clinicians prescribe in the face of diagnostic uncertainty or fear that a patient will not follow up if a condition worsens.^42,43^

Improving how antibiotics are used is critical for reducing antimicrobial resistance.^44,45^ In outpatient settings, key strategies focus on improving antibiotic decision-making and communication during clinical encounters; these strategies seek to optimize care once families have already arrived at a site of care and could help further improve guideline concordance in both in-person and telemedicine settings.^44,45^ A growing body of literature suggests that an alternative, complementary strategy is to support families in choosing to receive care at sites that are more judicious in their antibiotic use.^12,13,14,39,40,41,46,47,48,49^ This study suggests that supporting primary care practices so that they can offer telemedicine visits, as well as guiding families so that they choose primary care telemedicine instead of less judicious sites, may help reduce a child’s exposure to unnecessary antibiotics.

Our measure of guideline concordance indicates that antibiotic management in both visit modalities was generally congruent with the diagnosis provided, but that the diagnosis mix varied significantly. Among in-person visits, slightly more than half of visits received a viral diagnosis, and acute otitis media accounted for an additional quarter of visits. Among the telemedicine sample, two-thirds of visits received a viral diagnosis, and sinusitis was the most common bacterial diagnosis (accounting for 14.7% of visits). Several factors could contribute to these different rates of diagnosis, which could reflect either true differences in underlying case mix or biases in diagnoses driven by visit modality. If presenting children truly have the same underlying case mix, overdiagnosis of otitis media at in-person settings or of sinusitis via telemedicine could contribute to this pattern of diagnoses.^23^ Otitis media diagnosis requires visualization of the tympanic membrane, which is difficult in telemedicine encounters in the absence of specialized devices; sinusitis, in contrast, is diagnosed largely on history. Alternatively, presenting children could have a different underlying case mix at in-person vs telemedicine visits if families are self-triaging or if clinics are triaging to each site of care. Compared with virtual-only telemedicine sites, primary care may be in a better position to support appropriate clinical sorting since they offer both visit types and have the ability to convert a telemedicine to an in-person visit when needed.

To understand whether different rates of diagnoses between telemedicine and in-person visits is associated with signals of missed diagnoses, we complemented this analysis with assessment of follow-up visits and antibiotics. We did not observe significant differences in subsequent visits or antibiotics by visit modality in the 14 days after the initial visit. Thus, while primary care telemedicine visits had fewer bacterial diagnoses and fewer antibiotic prescriptions, patterns of follow-up care do not indicate missed diagnoses that warranted subsequent treatment.

### Limitations

Study limitations include that we are limited to data observable within the EHR. We do not have an objective assessment of child diagnosis outside the diagnosis provided by the clinician. For this reason, we also examined patterns of follow-up care to monitor for possible missed diagnoses or delayed treatment occurring in one site more than another. Additionally, because emergency department and urgent care use was not available, our analysis reports follow-up only within primary care settings. However, we note that after primary care telemedicine visits, our observed follow-up primary care visit rates were similar to follow-up visit rates in a prior claims analysis, which would have captured all sites of follow-up care.^14^ We also lack data on the nature of antibiotic stewardship programs within different practices or on the availability of teledevices (eg, otoscopes for remote evaluation) in the homes of families completing visits. Additionally, the nature of the EHR data did not allow us to distinguish reliably between audio-only vs audio-video visits. While we used IPTW to balance measured covariates, this design cannot account for unmeasured confounding. Residual confounding from unmeasured factors may remain. We note that use of telemedicine for ARTIs was low in these data, with 2% of all ARTI visits at these primary care practices occurring via telemedicine. Monitoring quality of antibiotic prescribing with evolving use of telemedicine will be important to maintain high-quality management.

## Conclusion

In this cross-sectional study of 694 primary care practices caring for children, telemedicine integrated within primary care was associated with judicious antibiotic prescribing without increased need for follow-up visits or antibiotic prescribing in the subsequent 2 weeks, all suggestive of high-quality care for ARTIs. Supporting primary care practices in providing telemedicine for acute care, such as for ARTIs, may be a way to promote antibiotic stewardship while providing ease of access for families.

## Notes

Supplement 1. **eTable 1.** ICD-10 Codes Used to Identify ARTI Visits **eFigure 1.** Love Plot Illustrating Covariate Balance Before and After Propensity Score Adjustment **eFigure 2.** Distribution of Propensity Score Before and After Propensity Score Adjustment **eFigure 3.** Distribution of Prognosis Score Before and After Propensity Score Adjustment **eFigure 4.** Stratified Analyses, Distribution of Propensity Score Before and After Propensity Score Adjustment **eTable 2.** Primary Care ARTI Index Visit Outcomes by Diagnosis and Visit Modality

Supplement 2.
Data Sharing Statement

## References

[1] Schweiberger K, Patel SY, Mehrotra A, Ray KN. Trends in pediatric primary care visits during the coronavirus disease of 2019 pandemic. Acad Pediatr. 2021;21(8):1426-1433. doi:10.1016/j.acap.2021.04.031 33984496 PMC8561008

[2] Hersh AL, Shapiro DJ, Pavia AT, Shah SS. Antibiotic prescribing in ambulatory pediatrics in the United States. Pediatrics. 2011;128(6):1053-1061. doi:10.1542/peds.2011-1337 22065263

[3] Kronman MP, Zhou C, Mangione-Smith R. Bacterial prevalence and antimicrobial prescribing trends for acute respiratory tract infections. Pediatrics. 2014;134(4):e956-e965. doi:10.1542/peds.2014-0605 25225144

[4] Donnelly JP, Baddley JW, Wang HE. Antibiotic utilization for acute respiratory tract infections in U.S. emergency departments. Antimicrob Agents Chemother. 2014;58(3):1451-1457. doi:10.1128/AAC.02039-13 24342652 PMC3957838

[5] Fleming-Dutra KE, Hersh AL, Shapiro DJ, . Prevalence of inappropriate antibiotic prescriptions among US ambulatory care visits, 2010-2011. JAMA. 2016;315(17):1864-1873. doi:10.1001/jama.2016.4151 27139059

[6] Burns SK, Krishnamurti T, Doan TT, Kahn JM, Ray KN. Parent care-seeking decisions for pediatric acute respiratory tract infections in the United States: a mental models approach. Acad Pediatr. 2023;23(7):1326-1336. doi:10.1016/j.acap.2023.02.011 36871609 PMC10475487

[7] Burns SK, Krishnamurti T, Doan TT, . Parent perceptions of telemedicine for acute pediatric respiratory tract infections: sequential mixed methods study. JMIR Pediatr Parent. 2024;7:e49170. doi:10.2196/49170 38227360 PMC10828946

[8] Shulman ST, Bisno AL, Clegg HW, . Clinical practice guideline for the diagnosis and management of group A streptococcal pharyngitis: 2012 update by the Infectious Diseases Society of America. Clin Infect Dis. 2012;55(10):1279-1282. doi:10.1093/cid/cis847 23091044

[9] Wald ER, Applegate KE, Bordley C, ; American Academy of Pediatrics. Clinical practice guideline for the diagnosis and management of acute bacterial sinusitis in children aged 1 to 18 years. Pediatrics. 2013;132(1):e262-e280. doi:10.1542/peds.2013-1071 23796742

[10] Lieberthal AS, Carroll AE, Chonmaitree T, . The diagnosis and management of acute otitis media. Pediatrics. 2013;131(3):e964-e999. doi:10.1542/peds.2012-3488 23439909

[11] Ray KN, Shi Z, Poon SJ, Uscher-Pines L, Mehrotra A. Use of commercial direct-to-consumer telemedicine by children. Acad Pediatr. 2019;19(6):665-669. doi:10.1016/j.acap.2018.11.016 30639759 PMC6620157

[12] Ray KN, Shi Z, Gidengil CA, Poon SJ, Uscher-Pines L, Mehrotra A. Antibiotic prescribing during pediatric direct-to-consumer telemedicine visits. Pediatrics. 2019;143(5):e20182491. doi:10.1542/peds.2018-2491 30962253 PMC6565339

[13] Ray KN, Martin JM, Wolfson D, . Antibiotic prescribing for acute respiratory tract infections during telemedicine visits within a pediatric primary care network. Acad Pediatr. 2021;21(7):1239-1243. doi:10.1016/j.acap.2021.03.008 33741531

[14] Wittman SR, Hoberman A, Mehrotra A, Sabik LM, Yabes JG, Ray KN. Antibiotic receipt for pediatric telemedicine visits with primary care vs direct-to-consumer vendors. JAMA Netw Open. 2024;7(3):e242359. doi:10.1001/jamanetworkopen.2024.2359 38483387 PMC10940962

[15] von Elm E, Altman DG, Egger M, Pocock SJ, Gøtzsche PC, Vandenbroucke JP; STROBE Initiative. The Strengthening the Reporting of Observational Studies in Epidemiology (STROBE) statement: guidelines for reporting observational studies. Lancet. 2007;370(9596):1453-1457. doi:10.1016/S0140-6736(07)61602-X 18064739

[16] Wittman SR, Yabes JG, Sabik LM, Kahn JM, Ray KN. Patient and family factors associated with use of telemedicine visits for pediatric acute respiratory tract infections, 2018-2019. Telemed J E Health. 2023;29(1):127-136. doi:10.1089/tmj.2022.0097 35639360 PMC9918348

[17] Shi Z, Mehrotra A, Gidengil CA, Poon SJ, Uscher-Pines L, Ray KN. Quality of care for acute respiratory infections during direct-to-consumer telemedicine visits for adults. Health Aff (Millwood). 2018;37(12):2014-2023. doi:10.1377/hlthaff.2018.05091 30633682 PMC6739118

[18] Wittman SR, Martin JM, Mehrotra A, Ray KN. Antibiotic receipt during outpatient visits for COVID-19 in the US, from 2020 to 2022. JAMA Health Forum. 2023;4(2):e225429. doi:10.1001/jamahealthforum.2022.5429 36800196 PMC9938423

[19] Kronman MP, Gerber JS, Grundmeier RW, . Reducing antibiotic prescribing in primary care for respiratory illness. Pediatrics. 2020;146(3):e20200038. doi:10.1542/peds.2020-0038 32747473 PMC7461202

[20] Yeramosu D, Kwok F, Kahn JM, Ray KN. Validation of use of billing codes for identifying telemedicine encounters in administrative data. BMC Health Serv Res. 2019;19(1):928. doi:10.1186/s12913-019-4753-2 31796039 PMC6892196

[21] Larson AE, Stange KC, Heintzman J, . Identifying virtual care modality in electronic health record data. Learn Health Syst. 2024;8(Suppl 1):e10411. doi:10.1002/lrh2.10411 38883878 PMC11176566

[22] Ralston SL, Lieberthal AS, Meissner HC, ; American Academy of Pediatrics. Clinical practice guideline: the diagnosis, management, and prevention of bronchiolitis. Pediatrics. 2014;134(5):e1474-e1502. doi:10.1542/peds.2014-2742 25349312

[23] Martinez KA, Rood M, Rothberg MB. Coding bias in respiratory tract infections may obscure inappropriate antibiotic use. J Gen Intern Med. 2019;34(6):806-808. doi:10.1007/s11606-018-4823-x 30652274 PMC6544729

[24] Centers for Disease Control and Prevention. Measurement and evaluation approaches to improve outpatient antibiotic prescribing in health systems. 2023. Accessed February 9, 2026. https://www.cdc.gov/antibiotic-use/pdfs/measurement-evaluation-improve-outpatient-508.pdf

[25] Feinstein JA, Hall M, Davidson A, Feudtner C. Pediatric Complex Chronic Condition System Version 3. JAMA Netw Open. 2024;7(7):e2420579. doi:10.1001/jamanetworkopen.2024.20579 39008301 PMC11250371

[26] Frost HM, McLean HQ, Chow BDW. Variability in antibiotic prescribing for upper respiratory illnesses by provider specialty. J Pediatr. 2018;203:76-85.e8. doi:10.1016/j.jpeds.2018.07.044 30195553

[27] Gerber JS, Prasad PA, Localio AR, . Racial differences in antibiotic prescribing by primary care pediatricians. Pediatrics. 2013;131(4):677-684. doi:10.1542/peds.2012-2500 23509168 PMC9923585

[28] Goyal MK, Johnson TJ, Chamberlain JM, ; Pediatric Care Applied Research Network (PECARN). Racial and ethnic differences in antibiotic use for viral illness in emergency departments. Pediatrics. 2017;140(4):e20170203. doi:10.1542/peds.2017-0203 28872046 PMC5613999

[29] Hersh AL, Shapiro DJ, Pavia AT, Fleming-Dutra KE, Hicks LA. Geographic variability in diagnosis and antibiotic prescribing for acute respiratory tract infections. Infect Dis Ther. 2018;7(1):171-174. doi:10.1007/s40121-017-0181-y 29273976 PMC5840100

[30] Fleming-Dutra KE, Demirjian A, Bartoces M, Roberts RM, Taylor TH Jr, Hicks LA. Variations in antibiotic and azithromycin prescribing for children by geography and specialty—United States, 2013. Pediatr Infect Dis J. 2018;37(1):52-58. doi:10.1097/INF.0000000000001708 28746259 PMC6622452

[31] *Cobalt: Covariate Balance Tables and Plots*. R package, version 4.6.1. [computer program]. Griefer, N; 2025. Accessed January 29, 2026. https://cran.r-project.org/web/packages/cobalt/index.html

[32] Austin PC, Stuart EA. Moving towards best practice when using inverse probability of treatment weighting (IPTW) using the propensity score to estimate causal treatment effects in observational studies. Stat Med. 2015;34(28):3661-3679. doi:10.1002/sim.6607 26238958 PMC4626409

[33] Stuart EA, Lee BK, Leacy FP. Prognostic score-based balance measures can be a useful diagnostic for propensity score methods in comparative effectiveness research. J Clin Epidemiol. 2013;66(8)(suppl):e2610062. doi:10.1016/j.jclinepi.2013.01.013 23849158 PMC3713509

[34] Li F, Zaslavsky AM, Landrum MB. Propensity score weighting with multilevel data. Stat Med. 2013;32(19):3373-3387. doi:10.1002/sim.5786 23526267 PMC3710526

[35] Wolf ER, Richards A, Lavallee M, . Patient, provider, and health care system characteristics associated with overuse in bronchiolitis. Pediatrics. 2021;148(4):e2021051345. doi:10.1542/peds.2021-051345 34556548 PMC8830481

[36] El Feghaly RE, Sainz LE, Lee BR, ; For REDUCE (Reducing Differences in Urgent Care Encounters – Antibiotic Choice) Collaborative. Sociodemographic differences in treatment of acute respiratory infections in pediatric urgent cares. Infect Control Hosp Epidemiol. 2024;46(2):171-179. doi:10.1017/ice.2024.196 39623533 PMC13244395

[37] *dplyr: A Grammar of Data Manipulation*. R package, version 1.1.4.9000 [computer program]. Wickham H, François R, Henry L, Müller K, Vaughan D; 2025. Accessed January 29 2026. https://cran.r-project.org/web/packages/dplyr/index.html

[38] *Survey: Analysis of Complex Survey Samples*. R package, version 4.4 [computer program]. Lumley, T; 2024. Accessed January 29, 2026. https://cran.r-project.org/web/packages/survey/index.html

[39] Martinez KA, Deshpande A, Stanley E, Rothberg MB. Antibiotic prescribing for respiratory tract infections in urgent care: a comparison of in-person and virtual settings. Clin Infect Dis. 2025;80(1):7-13. doi:10.1093/cid/ciae396 39078065

[40] Li KY, Ngai KM, Genes N. Differences in antibiotic prescribing rates for telemedicine encounters for acute respiratory infections. J Telemed Telecare. 2024;30(3):570-573. doi:10.1177/1357633X221074503 35075936

[41] Martinez KA, Rood M, Jhangiani N, Boissy A, Rothberg MB. Antibiotic prescribing for respiratory tract infections and encounter length: an observational study of telemedicine. Ann Intern Med. 2019;170(4):275-277. doi:10.7326/M18-2042 30285078

[42] Szymczak JE, Hayes AA, Labellarte P, . Parent and clinician views on not using antibiotics for mild community-acquired pneumonia. Pediatrics. 2024;153(2):e2023063782. doi:10.1542/peds.2023-063782 38234215

[43] Horwood J, Cabral C, Hay AD, Ingram J. Primary care clinician antibiotic prescribing decisions in consultations for children with RTIs: a qualitative interview study. Br J Gen Pract. 2016;66(644):e207-e213. doi:10.3399/bjgp16X683821 26852795 PMC4758501

[44] Sanchez GV, Fleming-Dutra KE, Roberts RM, Hicks LA. Core elements of outpatient antibiotic stewardship. MMWR Recomm Rep. 2016;65(6):1-12. doi:10.15585/mmwr.rr6506a1 27832047

[45] Sanchez GV, Kabbani S, Tsay SV, . Antibiotic stewardship in outpatient telemedicine: adapting centers for disease control and prevention core elements to optimize antibiotic use. Telemed J E Health. 2024;30(4):951-962. doi:10.1089/tmj.2023.0229 37856146

[46] King LM, Tsay SV, Hicks LA, Bizune D, Hersh AL, Fleming-Dutra K. Changes in outpatient antibiotic prescribing for acute respiratory illnesses, 2011 to 2018. Antimicrob Steward Healthc Epidemiol. 2021;1(1):1-8. doi:10.1017/ash.2021.230 35923647 PMC9345578

[47] Palms DL, Hicks LA, Bartoces M, . Comparison of antibiotic prescribing in retail clinics, urgent care centers, emergency departments, and traditional ambulatory care settings in the United States. JAMA Intern Med. 2018;178(9):1267-1269. doi:10.1001/jamainternmed.2018.1632 30014128 PMC6142958

[48] El Feghaly RE, Herigon JC, Kronman MP, ; Sharing Antimicrobial Reports for Pediatric Stewardship OutPatient (SHARPS-OP) Collaborative. Benchmarking of outpatient pediatric antibiotic prescribing: results of a multicenter collaborative. J Pediatric Infect Dis Soc. 2023;12(6):364-371. doi:10.1093/jpids/piad039 37262431

[49] Hanmer J, Burns SK, Wittman SR, Doan TT, Krishnamurti T, Ray KN. Parent preferences for acute respiratory tract infection care. JAMA Netw Open. 2025;8(8):e2525904. doi:10.1001/jamanetworkopen.2025.25904 40779265 PMC12334952

