# Supplementary material for: Primary Care Telemedicine vs In-Person Antibiotic Prescribing for Pediatric Respiratory Tract Infections
Source: JAMA Netw Open. 2026 May 1;9(5):e2610062. doi: 10.1001/jamanetworkopen.2026.10062 (PMC13135210; doi:10.1001/jamanetworkopen.2026.10062)
Supplement: Supplement 1. — eTable 1. ICD-10 Codes Used to Identify ARTI Visits eFigure 1. Love Plot Illustrating Covariate Balance Before and After Propensity Score Adjustment eFigure 2. Distribution of Propensity Score Before and After Propensity Score Adjustment eFigure 3. Distribution of Prognosis Score Before and After Propensity Score Adjustment eFigure 4. Stratified Analyses, Distribution of Propensity Score Before and After Propensity Score Adjustment eTable 2. Primary Care ARTI Index Visit Outcomes by Diagnosis and Visit Modality [file jamanetwopen-e2610062-s001.pdf]

Supplemental Online Content

Ray KN, Wittman S, Kelly MK, Ramachandran J, et al. Primary care telemedicine vs in-person antibiotic prescribing for pediatric respiratory tract infections. *JAMA Netw Open*. 2026;
9(5):e2610062. doi:10.1001/jamanetworkopen.2026.10062

eTable 1. ICD-10 Codes Used to Identify ARTI Visits
eFigure 1. Love Plot Illustrating Covariate Balance Before and After Propensity Score Adjustment
eFigure 2. Distribution of Propensity Score Before and After Propensity Score Adjustment
eFigure 3. Distribution of Prognosis Score Before and After Propensity Score Adjustment
eFigure 4. Stratified Analyses, Distribution of Propensity Score Before and After Propensity Score Adjustment
eTable 2. Primary Care ARTI Index Visit Outcomes by Diagnosis and Visit Modality

This supplemental material has been provided by the authors to give readers additional information about their work.

© 2026 Ray KN et al. *JAMA Network Open*.

eTable 1: ICD-10 Codes used to identify ARTI visits

DIAGNOSIS CATEGORY	ICD-10 CODES
**STREPTOCOCCAL PHARYNGITIS**	J02.0, J03.00, and/or A38.n
**ACUTE OTITIS MEDIA**	H66.00n, H66.01n, H66.1n, H66.2n, H66.3n, H66.4n, H66.9n, and/or H67.n
**SINUSITIS**	J01.0n, J01.1n, J01.2n, J01.3n, J01.4n, J01.8n, and/or J01.9n
**VIRAL ARTI**	J00, J02.8, J02.9, J03.8n, J03.9n, J04.n, J05.n, J06.n, J12.n, H65.n, H68.n, and/or H69.n

Legend: ICD-10 codes used to identify acute respiratory tract infection (ARTI) visits. We excluded ARTI episodes which began with an index visit that was coded as a well-child visit (because well-child visits were almost entirely conducted in-person) or with a diagnosis of bronchiolitis, COVID-19, or influenza. We also excluded episodes which began with an index visit that had a co-diagnosis warranting antibiotics (e.g., urinary tract infection,) based on ICD-10 codes used in prior studies.[19] We coded diagnosis according to a hierarchy, such that visits were coded as streptococcal pharyngitis visits if relevant codes were present [J02.0, J03.0n, A38.n], with remaining visits coded as acute otitis media if those codes were present [H66.00-H66.93, H67.1n-H67.9n], and remaining visits coded as bacterial sinusitis if those codes were present [J01.00-J01.91]. The remaining visits were coded as viral ARTIs [J00, J02.8, J02.9, J03.8n, J03.9n, J04.n, J05.n, J06.n, J12.n, H65.n, H68.n, H69.n], with the most common diagnoses within this group including laryngitis, tonsilitis, and viral pharyngitis.

© 2026 Ray KN et al. *JAMA Network Open*.

eFigure 1: Love Plot illustrating covariate balance before and after propensity score adjustment

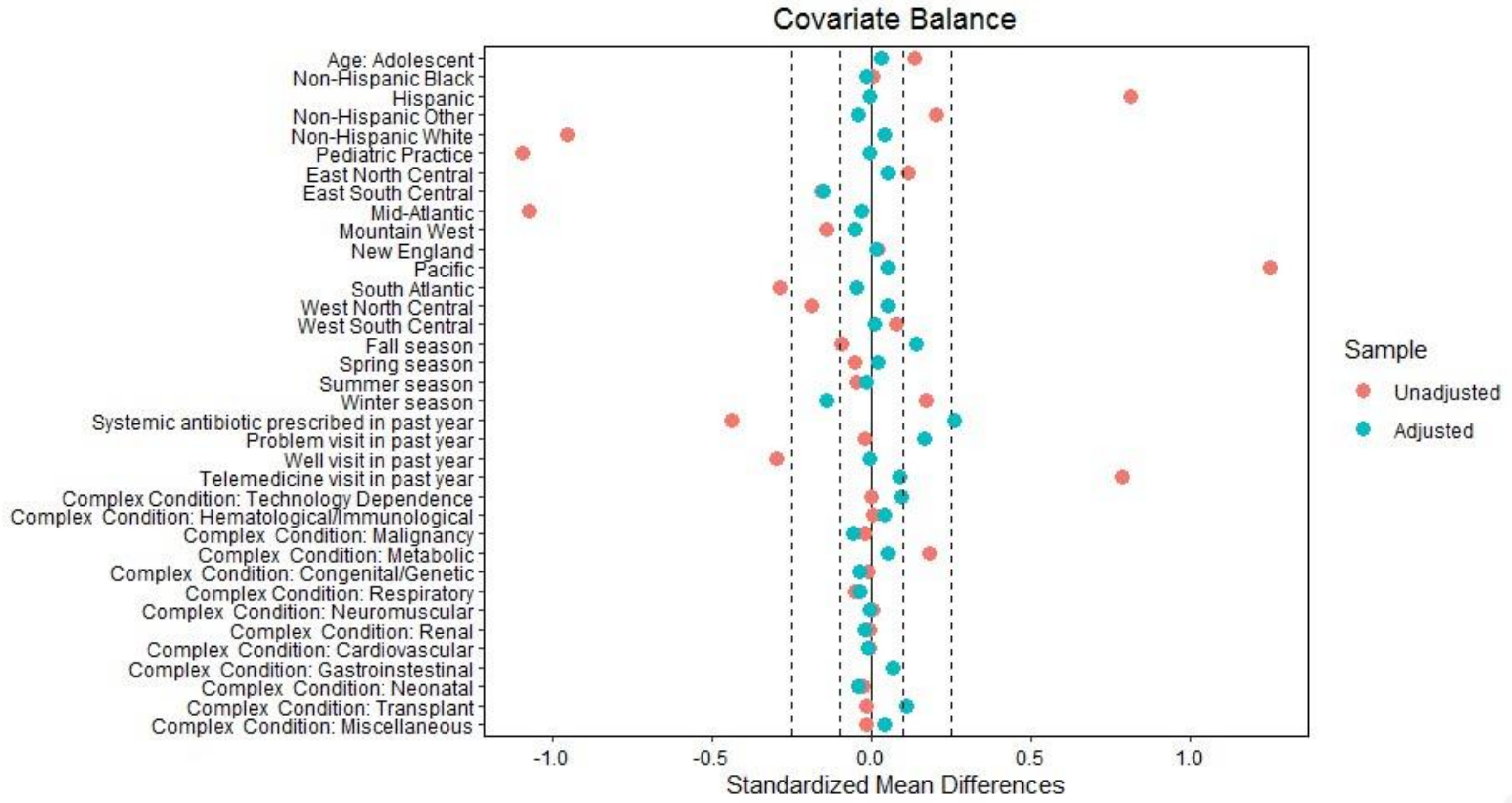

Legend: Love plot of the absolute standardized bias (ASB) before (red) and after (blue) the application of weights to assess covariate balance. Dashed lines closes to 0 represent a standardized mean difference of plus or minus 0.10; the dashed lines farther from 0 represent a standardized mean difference of plus or minus 0.25.

© 2026 Ray KN et al. *JAMA Network Open*.

eFigure 2: Distribution of propensity score before and after propensity score adjustment

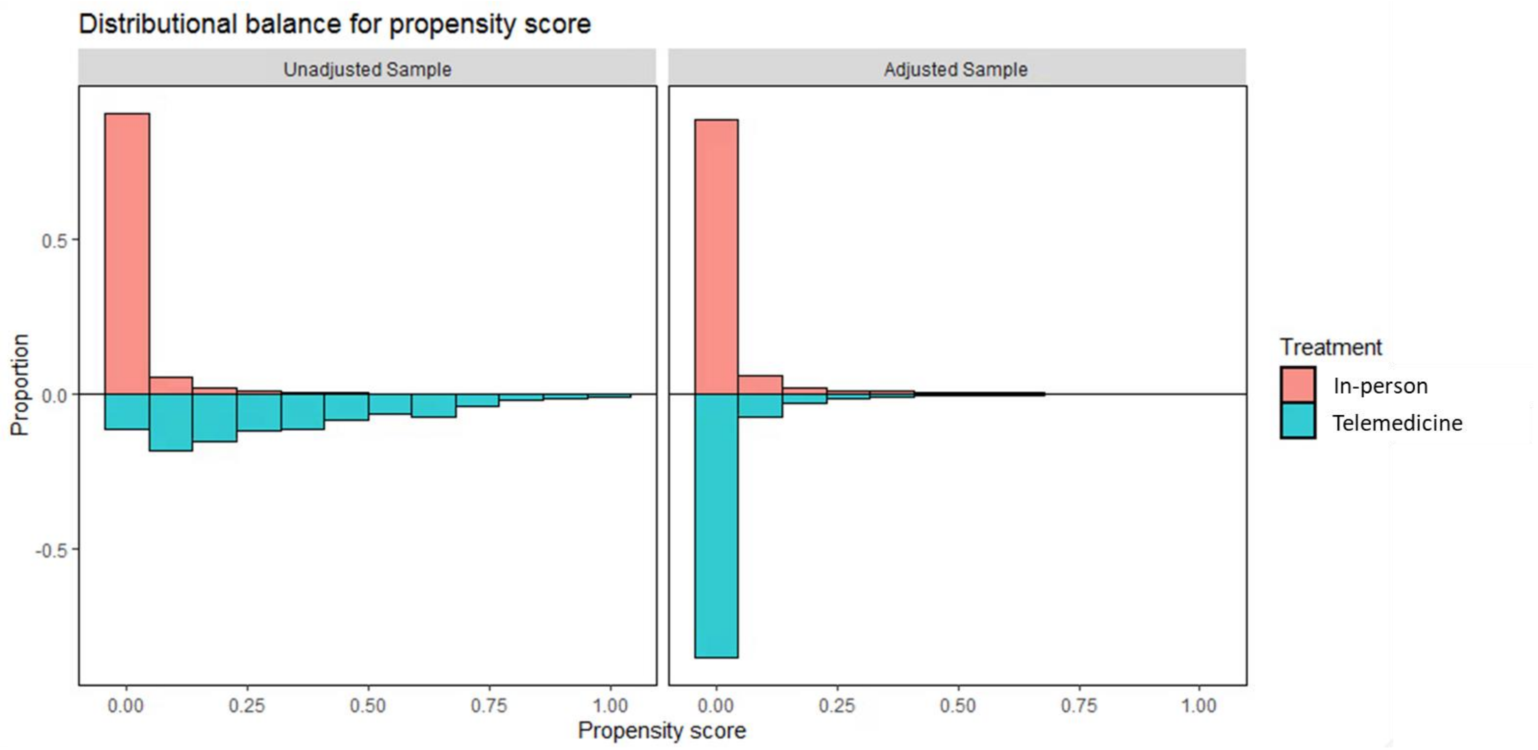

© 2026 Ray KN et al. *JAMA Network Open*.

eFigure 3: Distribution of prognosis score before and after propensity score adjustment

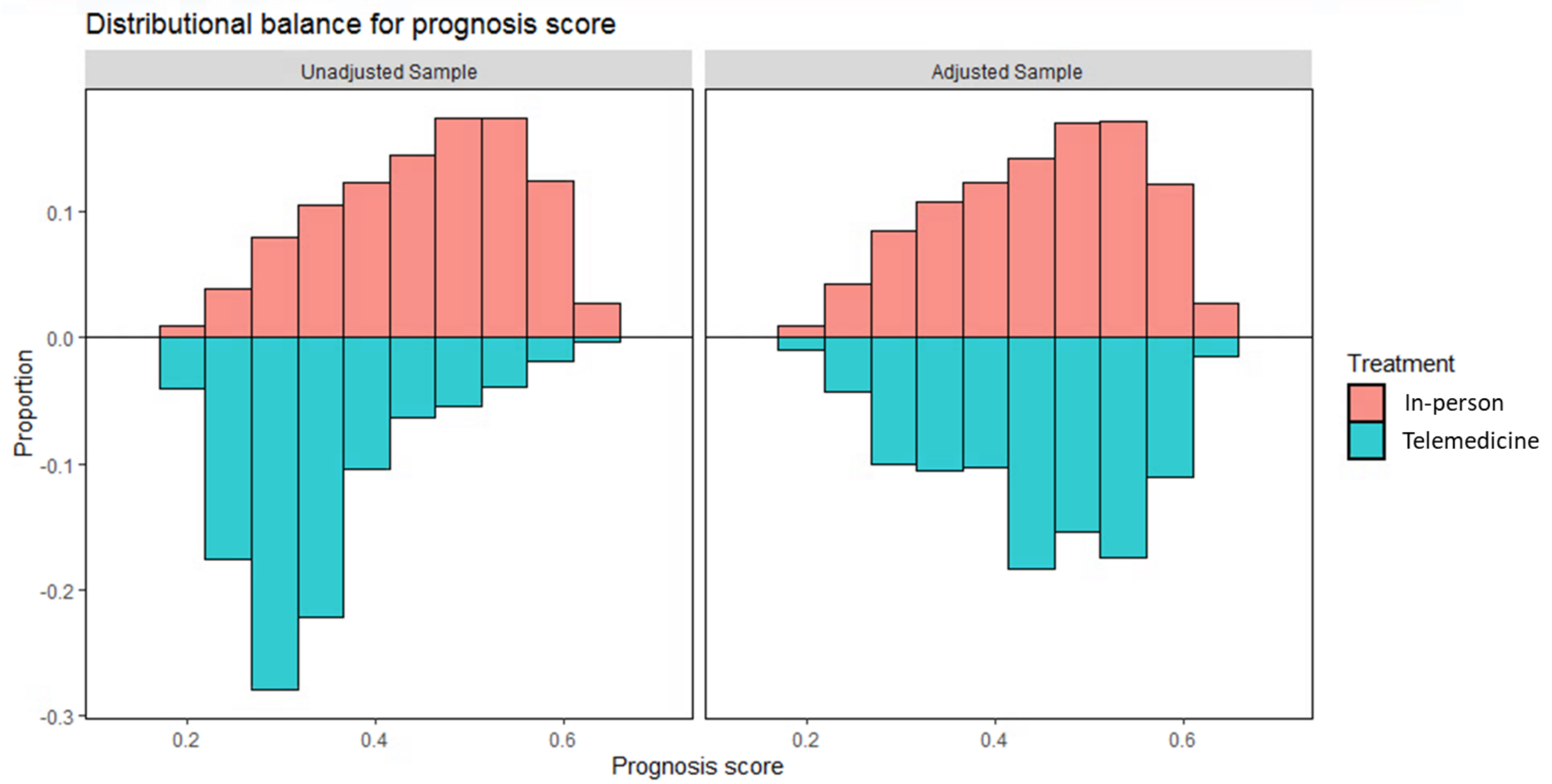

© 2026 Ray KN et al. *JAMA Network Open*.

eFigure 4: Stratified analyses, distribution of propensity score before and after propensity score adjustment

eFigure 4a: Propensity score distributions for child race and ethnicity stratified analyses

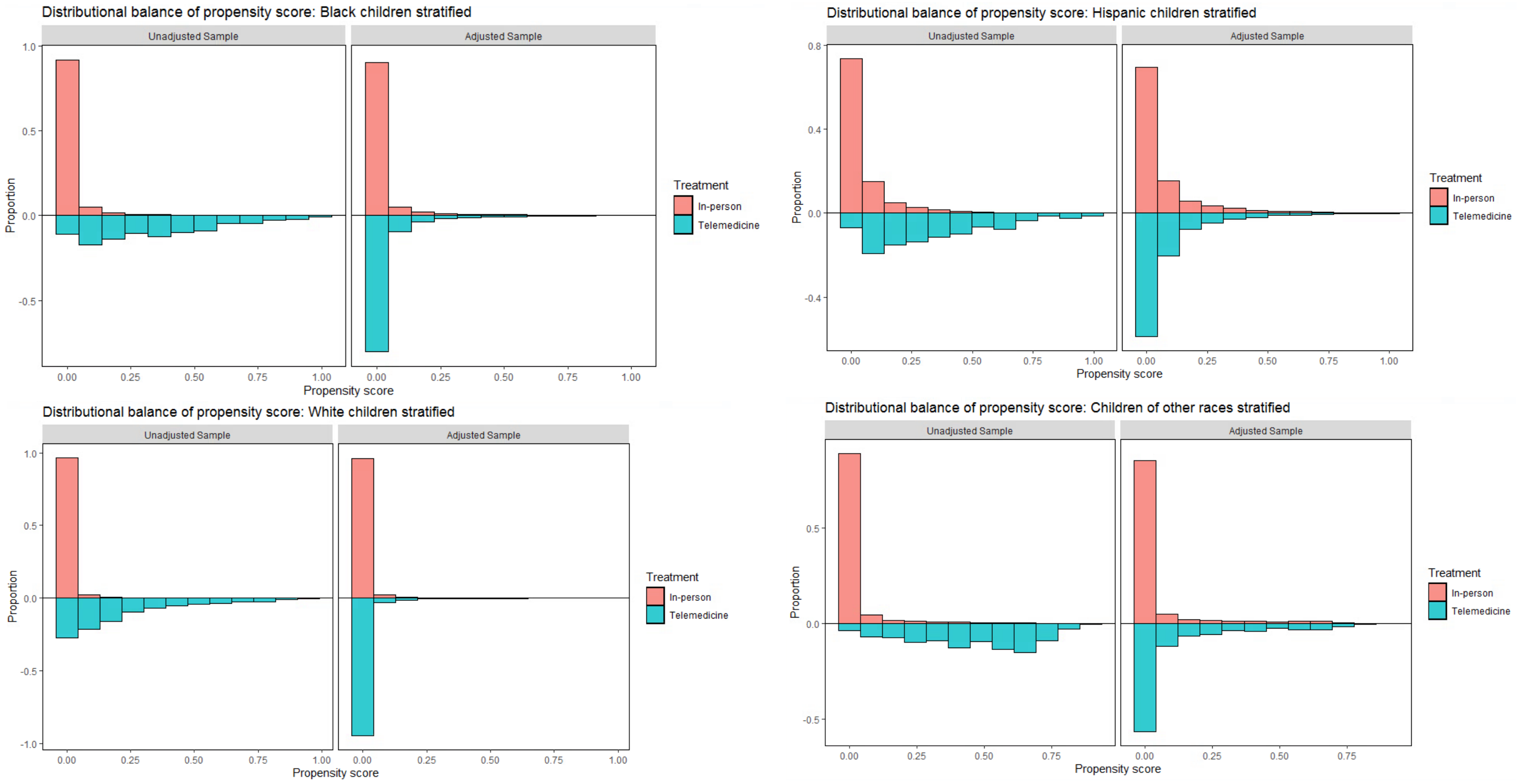

© 2026 Ray KN et al. *JAMA Network Open*.

eFigure 4b: Propensity score distributions for child age stratified analyses

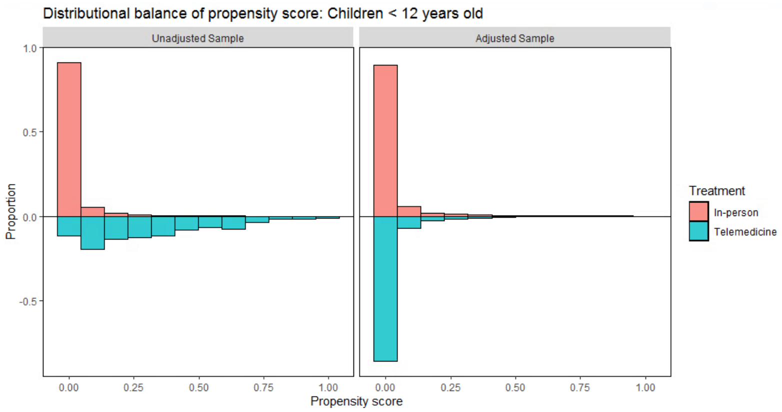

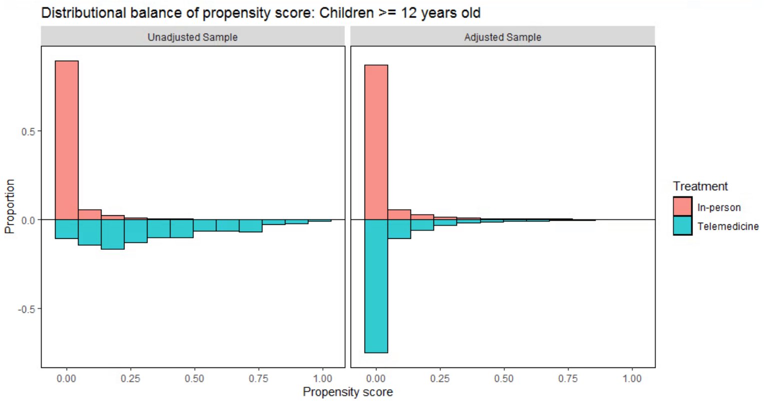

© 2026 Ray KN et al. *JAMA Network Open*.

eFigure 4c: Propensity score distributions for family language stratified analyses

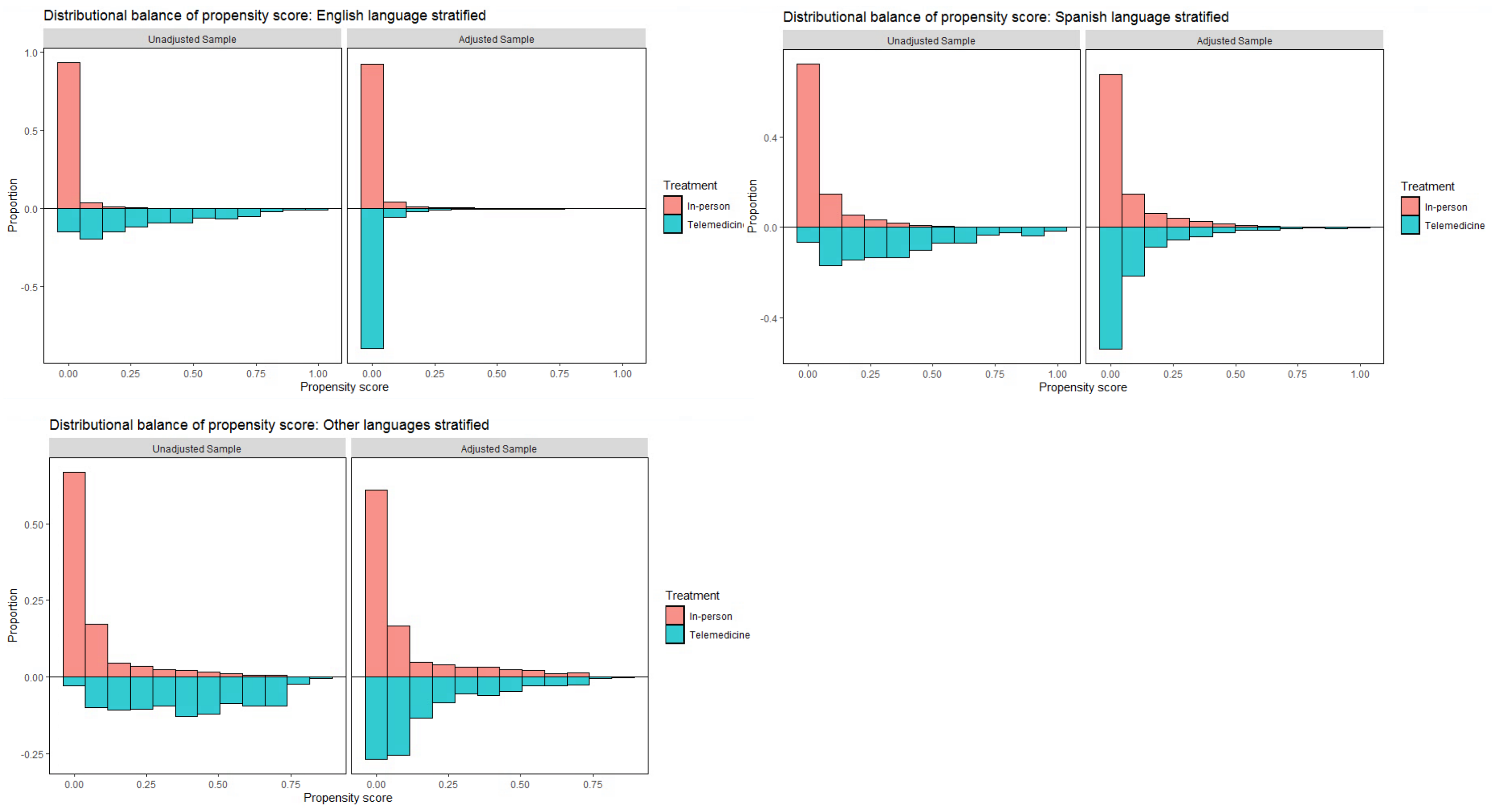

© 2026 Ray KN et al. *JAMA Network Open*.

eFigure 4d: Propensity score distributions for rural-urban stratified analyses

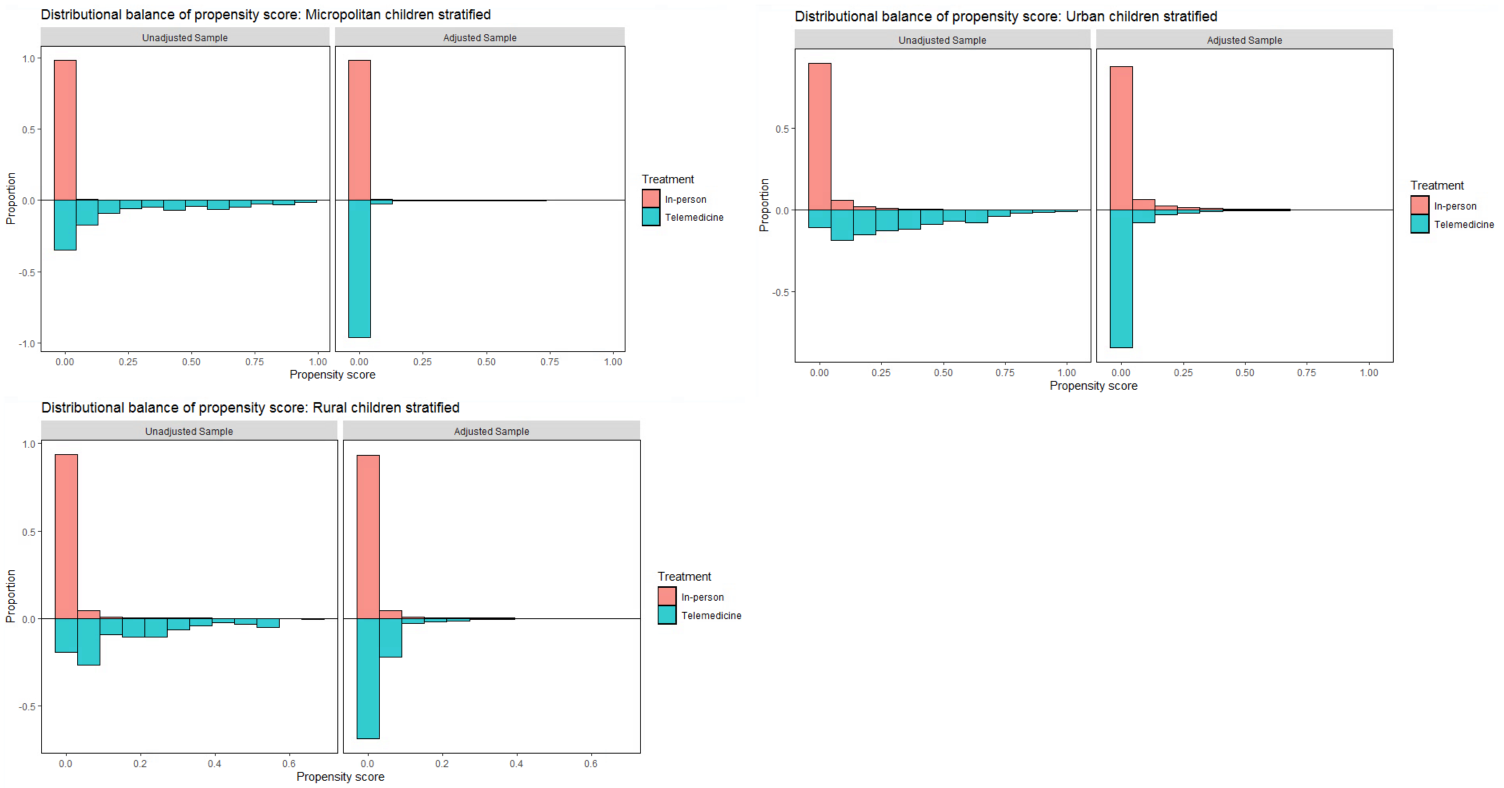

© 2026 Ray KN et al. *JAMA Network Open*.

eFigure 4e: Propensity score distributions for payer stratified analyses

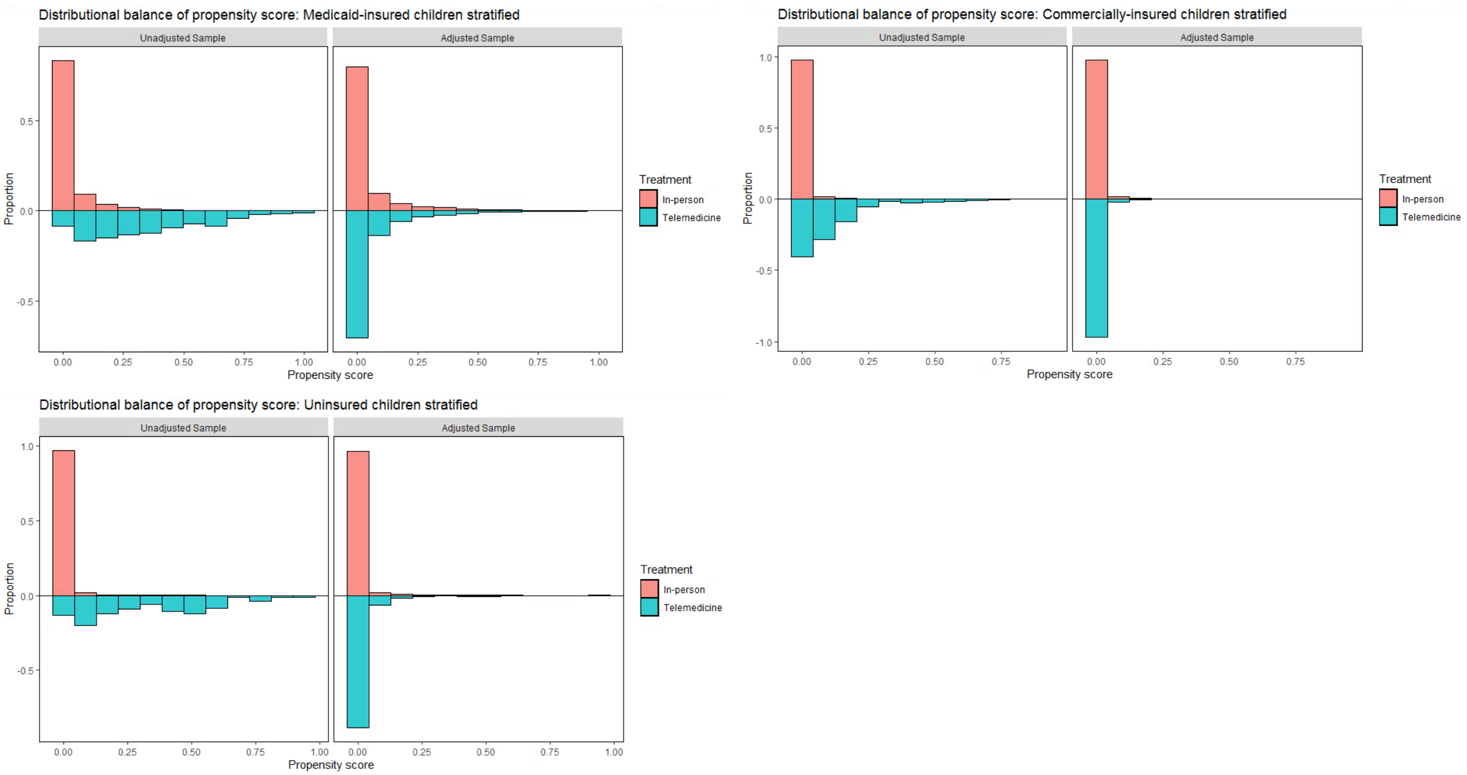

© 2026 Ray KN et al. *JAMA Network Open*.

eFigure 4f: Propensity score distributions for health system stratified analyses

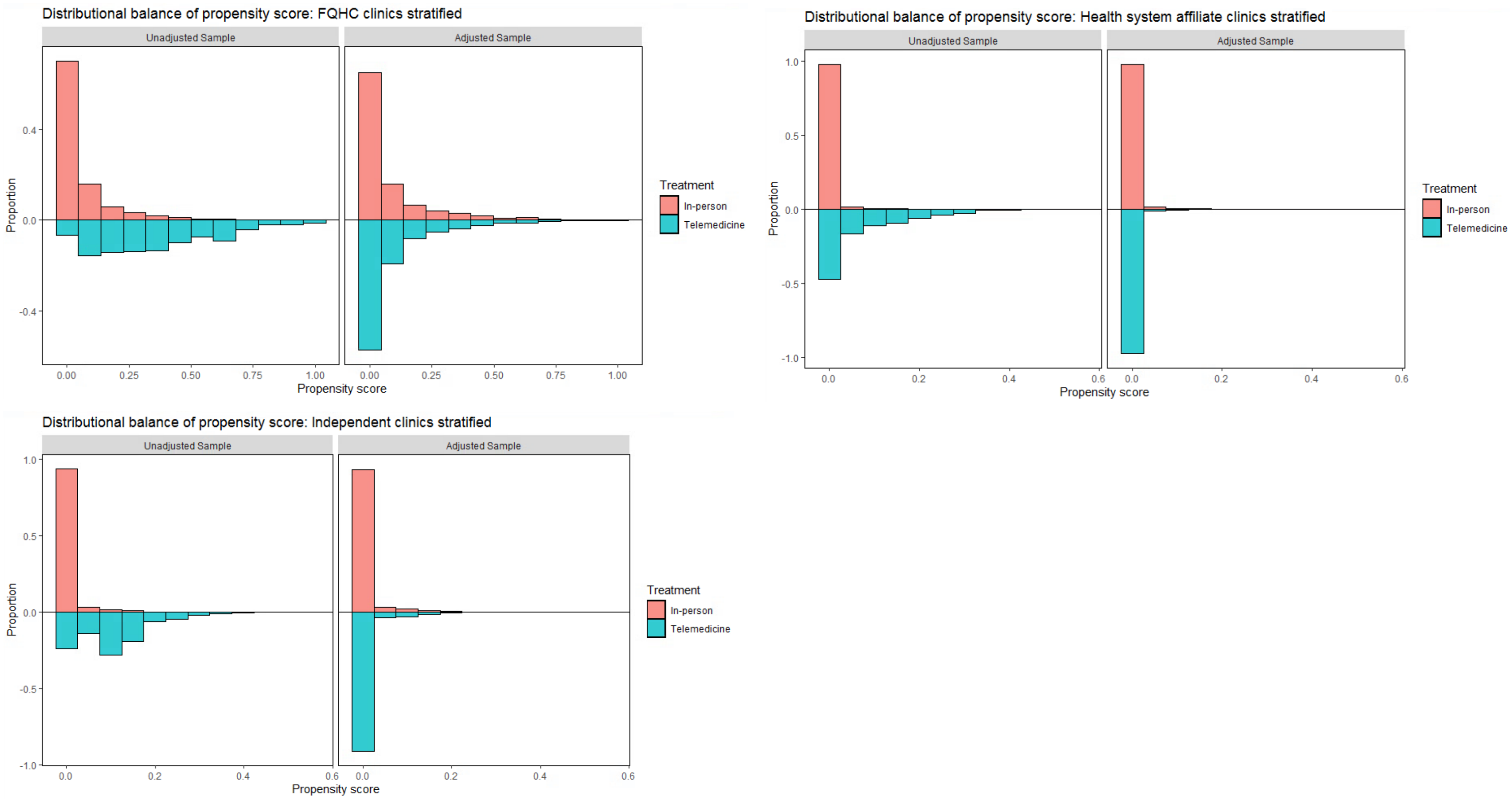

© 2026 Ray KN et al. *JAMA Network Open*.

eFigure 4g: Propensity score distributions for clinician specialty stratified analyses

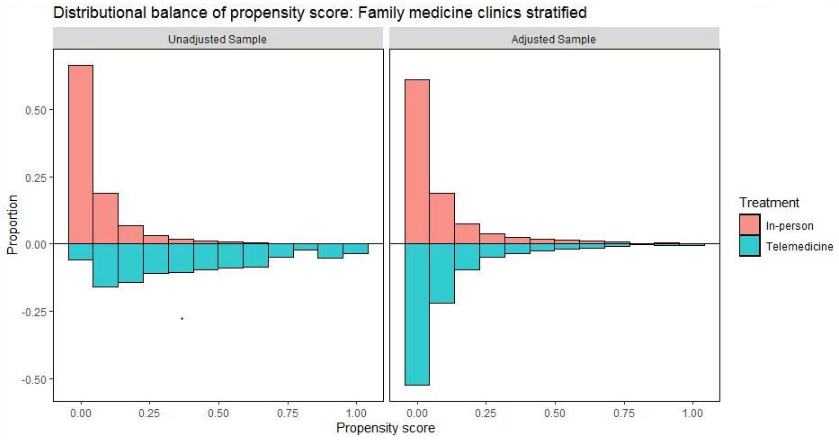

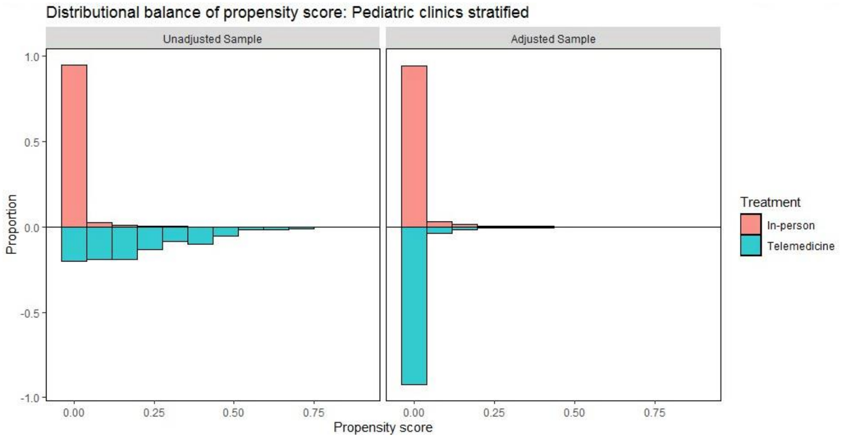

© 2026 Ray KN et al. *JAMA Network Open*.

eTable 2: Primary Care ARTI Index Visit Outcomes by Diagnosis and Visit Modality

	In-Person ARTI Visits, %	TM ARTI Visits, %	Average Treatment Effect (ATE), ∆%
**N, unweighted**	**438,148**	**11,482**	
	Weighted Percent (95 % CI)		∆% (95% CI)
**Received Each Diagnosis**			
- **Streptococcal Pharyngitis**	13.2 (12.5, 13.9)	7.3 (4.2, 10.4)	**-5.9 (-9.1, -2.7)**
- **Acute Otitis Media**	26.3 (25,3, 27.3)	11.0 (7.9, 14.1)	**-15.3 (-18.5, -12.1)**
- **Bacterial Sinusitis**	4.9 (4.1, 5.6)	14.7 (9.0, 20.5)	**9.9 (4.2, 15.6)**
- **Viral ARTI**	55.6 (54.0-57.2)	66.9 (59.8-74.1)	**11.3 (4.5, 18.2)**
**Among those with each diagnosis, Prescribed Antibiotics:**			
- **Streptococcal Pharyngitis**	94.9 (94.2, 95.7)	76.2 (66.1, 86.3)	**-18.8 (-28.9, -8.6)**
- **Acute Otitis Media**	91.4 (90.6, 92.3)	71.1 (60.6, 81.6)	**-20.3 (-30.6, -10.0)**
- **Bacterial Sinusitis**	96.2 (95.4, 97.0)	97.8 (96.4, 99.2)	**1.6 (0.4, 2.8)**
- **Viral ARTI**	10.0 (9.1, 11.0)	10.6 (4.9, 16.4)	0.6 (-4.9, 6.2)
**Among those with each diagnosis, Prescribed Guideline-Concordant Antibiotic Management:**			
- **Streptococcal Pharyngitis**	75.1 (73.0, 77.2)	57.8 (44.0, 71.6)	**-17.3 (-31.0, -3.7)**
- **Acute Otitis Media**	84.9 (83.4, 86.4)	83.7 (74.9, 92.5)	-1.2 (-10.5, 8.1)
- **Bacterial Sinusitis**	79.2 (75.6, 82.8)	77.5 (66.7, 88.4)	-1.6 (-10.9, 7.7)
- **Viral ARTI**	90.0 (89.0, 90.9)	89.4 (83.6, 95.1)	-0.6 (-6.2, 4.9)

Legend: Index visit outcomes (and 95% confidence intervals) for ARTI care episodes by diagnosis that begin with an in-person vs. telemedicine (TM) index visit. After limiting the sample to episodes with index visit with a given diagnosis, inverse probability of treatment weights were generated and applied. Average treatment effect is calculated as the difference between the outcome for TM vs. in-person episode of care. Abbreviations: ARTI, acute respiratory tract infection; CI, confidence interval.

© 2026 Ray KN et al. *JAMA Network Open*.
