# Supplementary material for: Primary Care Telemedicine vs In-Person Antibiotic Prescribing for Pediatric Respiratory Tract Infections
Source: JAMA Netw Open. 2026 May 1;9(5):e2610062. doi: 10.1001/jamanetworkopen.2026.10062 (PMC13135210; doi:10.1001/jamanetworkopen.2026.10062)
Supplement: Supplement 2. — Data Sharing Statement [file jamanetwopen-e2610062-s002.pdf]

Data Sharing Statement

Ray. Primary Care Telemedicine vs In-Person Antibiotic Prescribing for Pediatric Respiratory Tract Infections. *JAMA Netw Open*. Published May 01, 2026.
doi:10.1001/jamanetworkopen.2026.10062

Data
Data available: Yes
Data types: Deidentified participant data, Data dictionary
How to access data: De-identified analytic data set will be made available upon request for research purposes to researchers who provide a methodologically sound proposal after funded aims are complete with IRB approval and appropriate protections of participant privacy in place. Access requests should be sent to kristin.ray@chp.edu.
When available: beginning date: 08-01-2028, end date: 08-01-2032

Supporting Documents
Document types: None

Additional Information
Who can access the data: Researchers who provide an approved methodologically sound proposal.
Types of analyses: Researchers who provide an approved methodologically sound proposal.
Mechanisms of data availability: Researchers who provide a methodologically sound proposal after funded aims are complete with IRB approval and appropriate protections of participant privacy in place.
